# Pure pancreatic hepatoid carcinoma: a surgical case report and literature review

**DOI:** 10.1186/s40792-019-0723-5

**Published:** 2019-11-29

**Authors:** Takahiro Tomino, Mizuki Ninomiya, Rumi Matono, Fumiya Narutomi, Yumi Oshiro, Kenji Watanabe, Daisuke Taniguchi, Sho Nishimura, Yoko Zaitsu, Yuichiro Kajiwara, Tomoyuki Yokota, Kazuhito Minami, Takashi Nishizaki

**Affiliations:** 1 Department of Surgery, Matsuyama Red Cross Hospital, 1, Bunkyo-cho, Matsuyama-shi, Ehime 790-8524 Japan; 2 Department of Diagnostic Pathology, Matsuyama Red Cross Hospital, Matsuyama-shi, Ehime Japan; 3 Department of Center for Liver and Biliary and Pancreatic Diseases, Matsuyama Red Cross Hospital, Matsuyama-shi, Ehime Japan

**Keywords:** Hepatoid carcinoma, Pancreatic cancer, Laparoscopic distal pancreatectomy, Hepatocyte paraffin 1, Polyclonal carcinoembryonic antigen

## Abstract

**Background:**

Hepatoid carcinoma (HC) is an extra-hepatic neoplasm that shares the morphological and immunohistochemical features of hepatocellular carcinoma. Pancreatic HC exists as either pure or combined type. Pure pancreatic HC is extremely rare, with only a few cases reported in the literature to date. Because of the rarity of pure pancreatic HC, its clinical features including incidence, behavior, and prognosis remain unclear. We herein report the case of a 56-year-old man who developed pure pancreatic HC treated with surgical resection. We also include a review of the existing literature.

**Case presentation:**

A 56-year-old male patient was admitted to our hospital after a pancreatic cyst was identified by abdominal ultrasonography on a comprehensive medical examination. Endoscopic ultrasound revealed a cystic mass measuring 13 mm in size in the pancreatic head and a low-density mass measuring 16 mm in size in the pancreatic tail, which was partially enhanced on contrast-enhanced ultrasound. Contrast-enhanced computed tomography (CT) revealed a branch duct type intraductal papillary mucinous neoplasm in the pancreatic head and an early enhanced nodule measuring approximately 10 mm in size in the pancreatic tail. Endoscopic ultrasound-guided fine-needle aspiration of the hypervascular tumor was performed. The hypervascular tumor was suspected to be a solid pseudopapillary neoplasm. Laparoscopic spleen-preserving distal pancreatectomy was performed. Histology was identical to hepatocellular carcinoma of the liver. Immunohistochemically, the tumor cells were positive for hepatocyte paraffin 1, and a canalicular pattern was confirmed on the polyclonal carcinoembryonic antigen staining. The patient was diagnosed with a moderately differentiated pancreatic HC. The patient was followed up without adjuvant chemotherapy, and there was no evidence of recurrence at 6 months post-operatively.

**Conclusions:**

We present a case of moderately differentiated pure pancreatic HC. For the accurate preoperative diagnosis of pure pancreatic HC, biopsy is preferred to cytology or preoperative imaging studies such as CT. The prognosis of pure pancreatic HC depends on its differentiation.

## Background

Hepatoid carcinoma (HC) is an extra-hepatic neoplasm that shares the morphological and immunohistochemical features of hepatocellular carcinoma. The first case of HC reported by Ishikura in 1985 was of a gastric neoplasm with these characteristics [1]. HC is a rare neoplasm that has been described in different organs such as the stomach, pancreas, lung, gallbladder, ovary, and colon [2–7]. With the most common location being the stomach, followed by the ovaries [8–13].

Several cases of pancreatic HC have been reported since pancreatic HC was first reported by Yano in 1999 [14]. Pancreatic HC exists as pure type or combined type. Pure pancreatic HC has been referred to as hepatoid adenocarcinoma, hepatoid carcinoma, and ectopic hepatocellular carcinoma [14–16]. Combined pancreatic HC refers to HC with other histological components such as neuroendocrine tumor, endocrine carcinoma, islet cell glucagonoma, or pancreatic ductal adenocarcinoma [17–19]. Pure pancreatic HC is extremely rare, with few cases having been reported to date in the English literature. Because of the rarity of pure pancreatic HC, its clinical features including the incidence, behavior, and prognosis remain unclear.

We herein report the case of a 56-year-old man who developed pure pancreatic HC and was treated with surgical resection. We also conducted a review of the previous literature.

## Case presentation

**Fig. 1:**
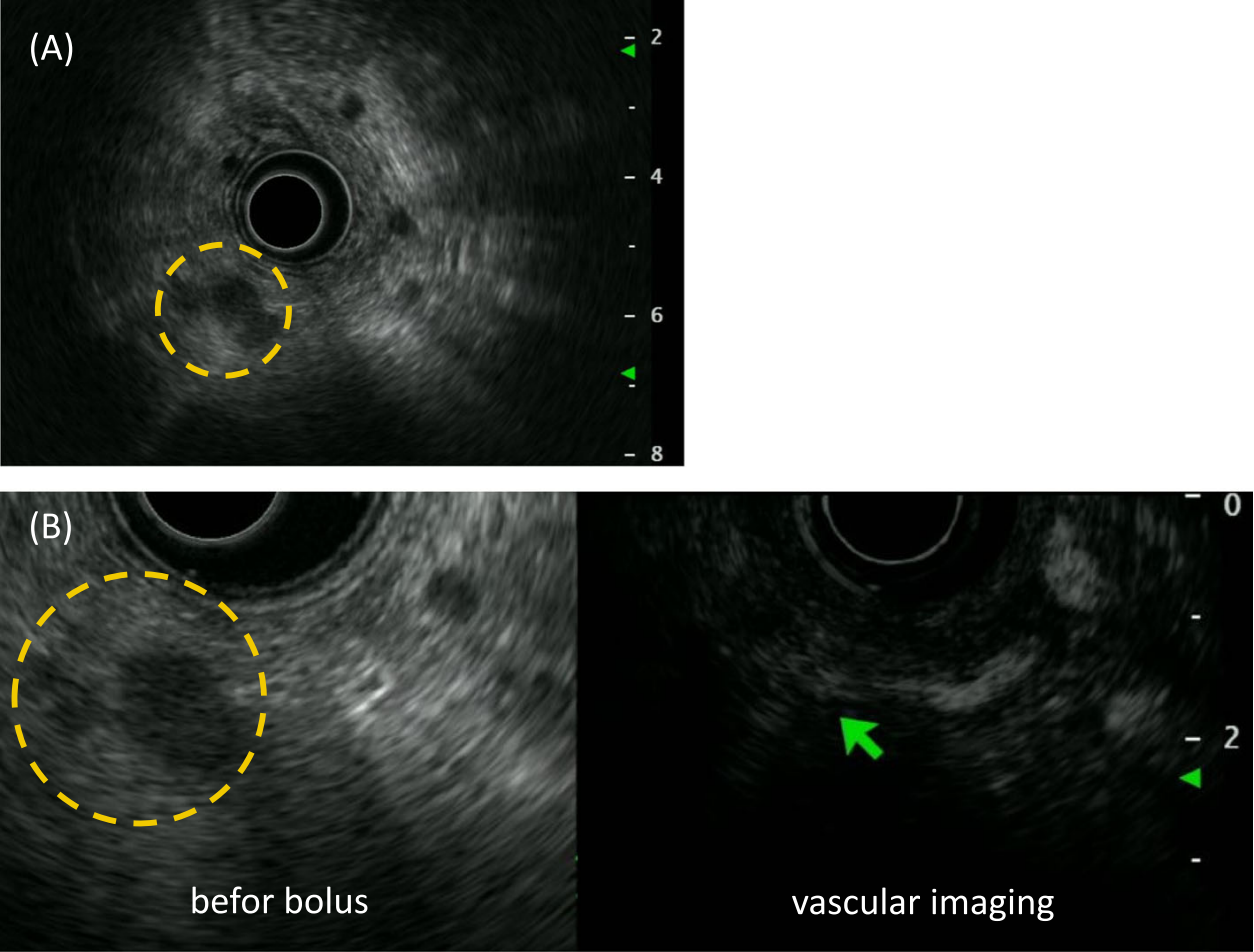
Endoscopic ultrasound findings. **a** Endoscopic ultrasound showed a low-density mass measuring about 16 mm in size in the pancreatic tail (circle). **b** The low-density mass in the pancreatic tail was partially enhanced on the contrast-enhanced ultrasound with bolus administration of Sonazoid

**Fig. 2:**
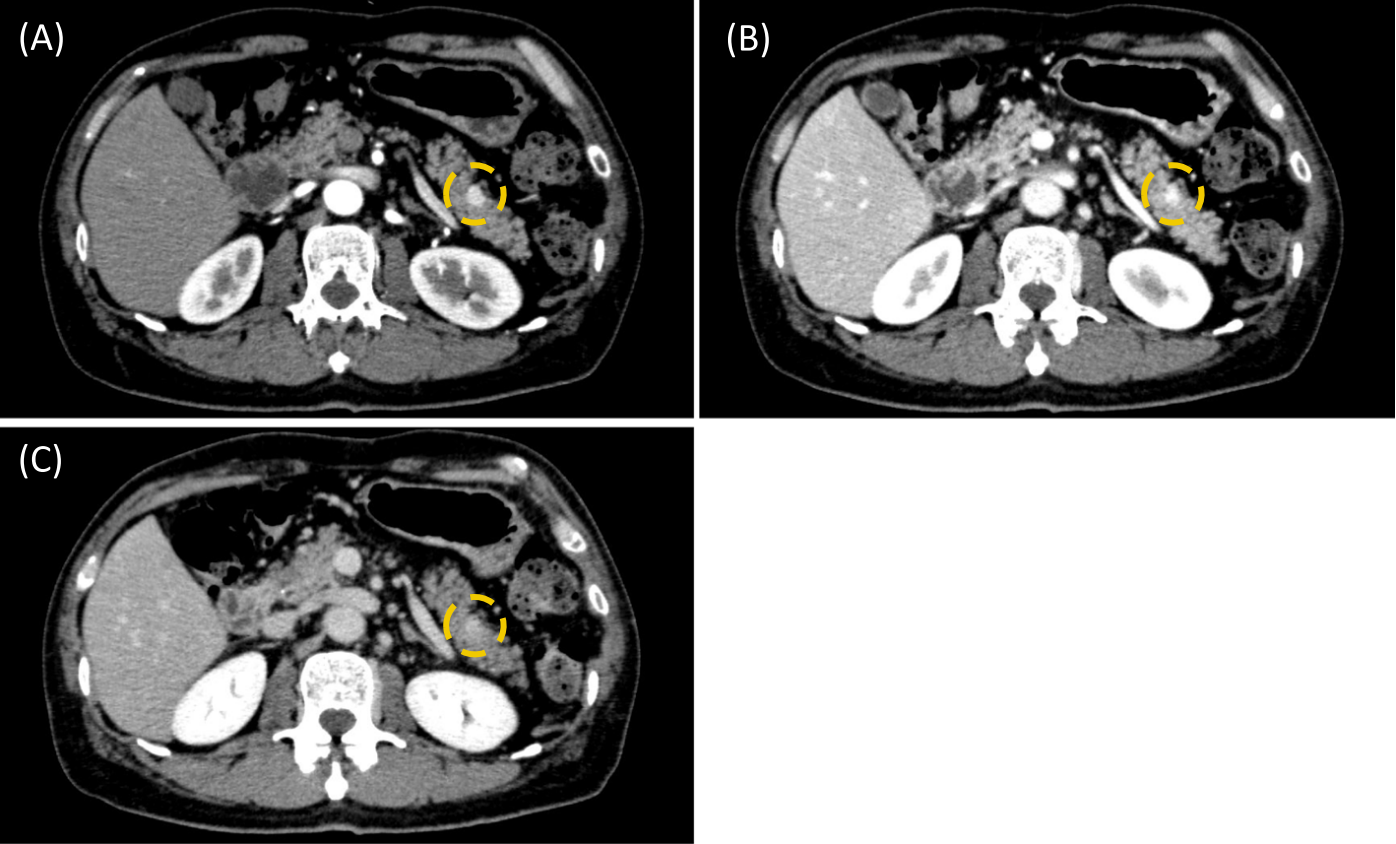
Computed tomography findings. Contrast-enhanced computed tomography showed an early enhanced nodule about 10 mm in size in the pancreatic tail (circle). An enhancement of the nodule lasted until late phase, although its density was gradually attenuated. **a** The arterial phase. **b** The portal phase. **c** The late phase

A 56-year-old male patient presented with a pancreatic cyst identified by abdominal ultrasonography on a comprehensive medical examination and was admitted to our hospital. He had a past medical history of type 2 diabetes, hyperlipidemia, and chronic hepatitis C for which he received interferon therapy for chronic hepatitis C more than 20 years previously. He had no family history of cancer. Laboratory tests revealed normal levels of carcinoembryonic antigen (CEA) and carbohydrate antigen 19–9, and alpha-fetoprotein (AFP) and protein induced by vitamin K absence or antagonist-II (PIVKA-II) were absent. Endoscopic ultrasound (EUS) showed a cystic mass measuring 13 mm in size in the pancreatic head and a low-density mass measuring 16 mm in size in the pancreatic tail (Fig. 1a), which was partially enhanced on the contrast-enhanced ultrasound image (Fig. 1b). Contrast-enhanced computed tomography (CT) revealed branch duct type intraductal papillary mucinous neoplasms in the pancreatic head and an early enhanced nodule measuring approximately 10 mm in size in the pancreatic tail (Fig. 2a–c). An enhancement of the nodule lasted until the late phase, although its density was gradually attenuated. Magnetic resonance imaging (MRI) did not detect the corresponding nodule in the pancreatic tail.

**Fig. 3:**
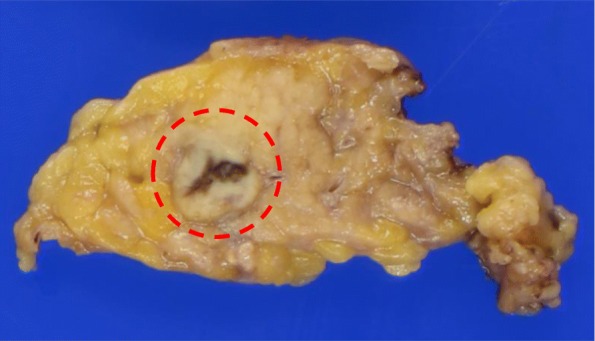
Macroscopic findings of the resected specimen. A well-circumscribed whitish-yellow solid mass, measuring 7 mm at the greatest dimension, was located in the pancreatic tail (circle)

**Fig. 4:**
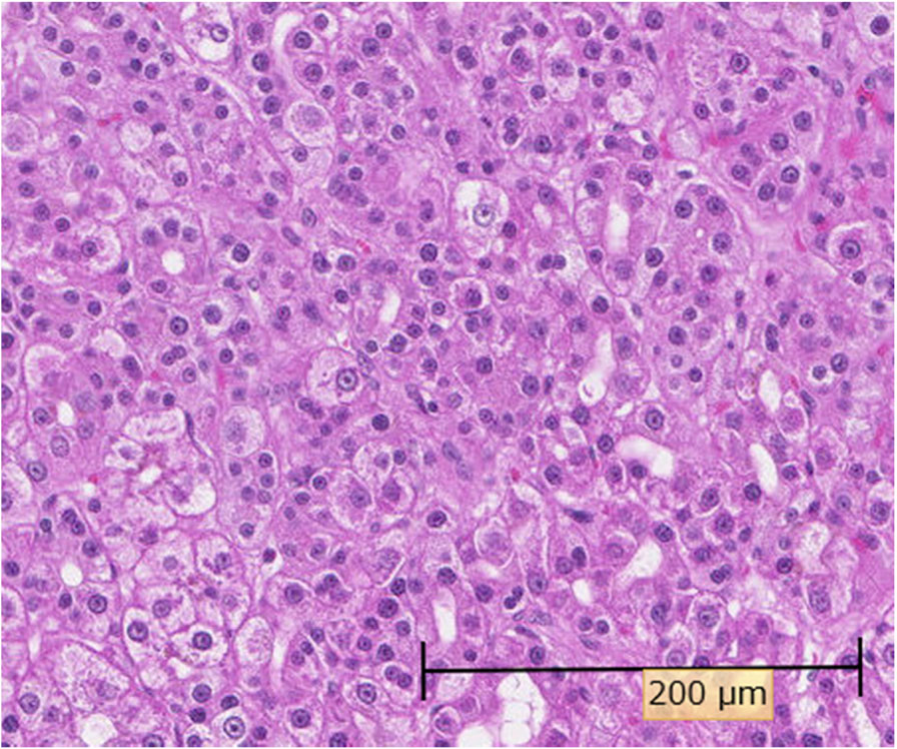
Hematoxylin and eosin staining findings. Polygonal tumor cells with round nuclei and abundant eosinophilic cytoplasm formed thick trabeculae in the tumor. The differentiation was moderate

**Fig. 5:**
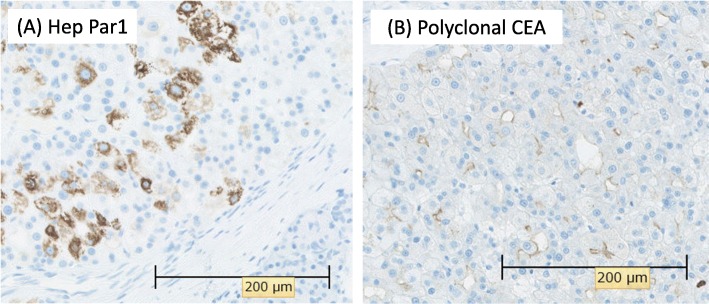
Immunohistochemical staining findings. **a** The tumor cells were positive for hepatocyte paraffin 1. **b** The tumor cells were positive for polyclonal carcinoembryonic antigen (CEA). A canalicular pattern was confirmed on polyclonal CEA immunohistochemical staining

Based on the above features, our initial differential was that of a neuroendocrine tumor or a solid pseudopapillary neoplasm (SPN). Endoscopic ultrasound-guided fine-needle aspiration (FNA) was performed to make a definitive diagnosis. FNA cytology showed that the tumor cells exhibited an acidophilic cytoplasm with small, round nuclei. Immunohistochemistry was performed to differentiate between a neuroendocrine tumor, SPN, and acinar cell carcinoma. The tumor cells were positive for cytokeratin, nuclear/membranous β-catenin, CD10, and CD56 and were negative for chromogranin A, synaptophysin, progesterone receptor, vimentin, and Bcl-10. Therefore, we suspected that the hypervascular tumor in the pancreatic tail was suspected to be SPN, but the results were not convincing. Laparoscopic spleen-preserving distal pancreatectomy was performed. Macroscopically, a well-circumscribed whitish-yellow solid mass, measuring 7 mm in the greatest dimension, was found in the pancreatic tail (Fig. 3). Histologically, polygonal tumor cells with round nuclei and abundant eosinophilic cytoplasm formed thick trabeculae. The differentiation was moderate (Fig. 4). Immunohistochemically, the tumor cells were positive for hepatocyte paraffin 1, AE1/AE3, and CD10 and negative for AFP, progesterone receptor, vimentin, chromogranin A, and synaptophysin (Fig. 5a). A canalicular pattern was confirmed on the polyclonal CEA staining (Fig. 5b). HC is characteristically hepatocyte paraffin 1 (HepPar1)-positive and has a canalicular pattern on polyclonal CEA staining. Finally, a diagnosis of moderately differentiated pancreatic HC was made. The patient’s postoperative course was uneventful, and he was discharged in good health 10 days after the operation. The patient did not receive adjuvant chemotherapy and remained recurrence-free at 6 months after the surgery. The serum levels of AFP (3 ng/mL) and PIVKA-II (28 mAU/mL) were normal at 1 month after the surgery. The latest serum levels of AFP (2 ng/mL) and PIVKA-II (25 mAU/mL) were normal at 6 months after the surgery.

## Discussion

Histologically, pancreatic HC can be categorized as either pure type or combined type. We conducted a systematic review of the English literature using the PubMed search engine and found 20 resected cases of pure pancreatic HC. The present case and 20 reported cases of resected pure pancreatic HC are summarized in Table 1 [28–32, 34–37].

**Table 1:** Reported surgical cases of pure pancreatic hepatoid carcinoma in the literature

Reference	Age	Gender	Location	Symptoms	Preoperative imaging study	Preoperative diagnostic method	Preoperative diagnosis	Postoperative pathological differentiation	Recurrence	Outcome
[14]	57	M	Head	Epigastric pain, vomiting, and fever	CT	None	Not listed	Well to moderate	Liver metastasis	Dead, 2.8 months
[28]	70	M	Body	None	CT	None	Not listed	Well	None	Alive, 12 months
[16]	51	M	Body and tail	Upper gastrointestinal hemorrhage	CT, EUS	Biopsy	Nondiagnostic tissue	Moderate	None	Alive, 14 months
[29]	32	M	Tail	Epigastric pain	CT	Biopsy	Hepatoid carcinoma	Well	None	Alive, 18 months
[26]	49	F	Tail	Body weight loss	US, CT, MRI, ERCP	Pancreatic juice cytology	Adenocarcinomatous cells	Well	Liver metastasis	Alive, 48 months
[15]	58	M	Body	Back and flank pain	CT	FNA	Hepatocytes	Well	None	Alive, 15 months
[11]	53	F	Body and tail	Epigastric pain	Not listed	None	Not listed	Moderate	Liver metastasis	Alive, 22 months
[12]	79	F	Body	None	CT	None	Not listed	Poor	Liver metastasis	Dead, 2 months
[30]	80	M	Head	Nausea, emesis, diarrhea, and weight loss	MRI	None	Not listed	Moderate	None	Alive, 8 months
[31]	69	M	Body and tail	Chest pain	CT, EUS	FNA	Hepatoid carcinoma	Moderate	None	Alive, 4 months
						Biopsy	Hepatoid carcinoma			
[32]	61	F	Tail	None	CT, PET-CT, EUS	FNA	Lymphoma or neuroendocrine tumor	Moderate	None	Alive, 60 months
[33]	57	M	Head	Jaundice	CT	None	Not listed	Moderate	None	Alive, 18 months
[34]	47	M	Tail	Groin pain and backache	US, CT, MRI	None	Not listed	Moderate	None	Alive, 8 months
[21]	59	M	Body	None	US, CT, MRI, EUS	FNA	SPN	Well	None	Alive, 12 months
[35]	61	M	Tail	None	MRI, EUS	FNA	Acinar cell carcinoma	Moderate	None	Alive, 6 months
[23]	67	M	Tail	None	CT, MRI	None	Not listed	Moderate	None	Alive, 6 months
[36]	56	M	Tail	None	US, CT, MRI	None	Not listed	Moderate	None	Alive, 36 months
[37]	78	M	Head	None	US, CT, MRI	Biopsy	Epithelial tumor	Well	None	Dead from acute heart attack, 2 months
[25]	83	M	Body	Abdominal pain	CT	Biopsy	Neuroendocrine tumor	Moderate	None	Alive, 107 months
	72	M	Tail	Back pain	US, CT	None	Not listed	Well	None	Dead from pulmonary embolism, 1 month
Present case	56	M	Tail	None	CT, MRI, EUS	FNA	SPN	Moderate	None	Alive, 6 months

*CT* computed tomography, *EUS* endoscopic ultrasound, *US* ultrasound, *MRI* magnetic resonance imaging, *ERCP* endoscopic retrograde cholangiopancreatography, *FNA* fine needle aspiration, *PET-CT* positron emission tomography-computed tomography, *SPN* solid pseudopapillary neoplasm

The median age of patients with resected pure pancreatic HC was 59 years (range 32–83), and most patients were men (81%). With regard to tumor location, most tumors (81%) were located in the body or tail of the pancreas, whereas approximately 75% of all pancreatic carcinomas occurred in the head or neck of the pancreas [20]. We found that 47% of the patients who had resectable pure pancreatic HC in the body or tail of the pancreas had no symptoms at the time of medical examination. Conversely, 75% of the patients with resectable pure pancreatic HC in the head of the pancreas had some symptoms. Clinically, the patients who had resectable pure pancreatic HC in the body or tail of the pancreas were less symptomatic than those with tumors located in the head of the pancreas.

The diagnosis of pure pancreatic HC based solely on preoperative imaging studies is difficult due to the non-specific radiologic features of this tumor [21]. In fact, none of the patients who underwent preoperative imaging studies such as CT received a precise diagnosis because of the radiographic characteristics with various vascular patterns in tumor enhancement. Therefore, preoperative imaging studies alone are insufficient to accurately diagnose pure pancreatic HC.

Immunohistochemical studies play an important role in making a definitive diagnosis. However, biopsy provides a more accurate diagnosis than cytology. In a series of 21 cases of resected pure pancreatic HC, a biopsy was performed in 5 cases and cytology was performed in 7 cases for preoperative workup. In the biopsy cases, 2 of 5 cases (40%) were positive for HepPar1 on immunohistochemical staining and were diagnosed with pancreatic HC. Conversely, 5 of 7 cytology cases (71%) were misdiagnosed as other cancers such as SPN. Considering that the overall 5-year survival rate of SPN is approximately 95% [22], and that of pancreatic HC is 40.4% [23], an accurate diagnosis of pancreatic HC is very important. This suggests that biopsy is essential for the preoperative diagnosis of pure pancreatic HC. In the present case, preoperative immunohistochemistry precluded correct diagnosis because of the small amount of the tissue and the under-recognition of this tumor. In patients with an accurate preoperative diagnosis of pure pancreatic HC, laparoscopic distal pancreatectomy might be a better choice of treatment, because some reports described regional lymph node metastasis on pathological review of resected specimens [12, 33].

While many hepatocellular carcinoma tumors express AFP and PIVKA-II at levels which may be detectable in the serum, preoperative AFP and PIVKA-II are not usually measured in patients with pancreatic HC. The rarity of this tumor makes it difficult to endorse the sensitivity of AFP and PIVKA-II as a screening test for pure pancreatic HC. Nevertheless, these may be useful markers for postoperative surveillance.

Previous reports have described that pancreatic HC usually has an aggressive clinical course and an extremely poor prognosis [11, 24]. Recently, Yang et al. [25] reported the prognosis of pancreatic HC. The authors described four histological subtypes of pancreatic HC, namely, with (1) pure HCC-like morphology, (2) neuroendocrine differentiation, (2) true glandular differentiation, and (4) acinar cell differentiation. Pure pancreatic HC was associated with better disease-specific survival than the other subtypes. The 5-year disease-specific survival rate of pure pancreatic HC was 77.3%, whereas that of pancreatic HC with neuroendocrine differentiation was 37.5% and that of pancreatic HC with true glandular differentiation or acinar cell differentiation was 0%. We re-reviewed the hematoxylin and eosin staining of 20 reported cases of resected pure pancreatic HC and assessed their differentiations. Among the resected cases, 7 cases were diagnosed as well-differentiated pure pancreatic HC, 13 cases were moderately differentiated, and 1 case was poorly differentiated. Excluding the sudden death cases, all the patients with well-differentiated pancreatic HC survived for more than 12 months after surgery. Contrarily, the poorly differentiated case had a short survival time of 2 months after surgery. Moreover, one patient with a poorly differentiated unresectable tumor who underwent chemotherapy (gemcitabine) died 3 months after the first consultation [9]. In brief, patients with poorly differentiated type tumors had a worse serious prognosis than did those with well or moderately differentiated type.

Recurrence after surgery was confirmed in 4 of 21 resected cases (19%), and in all cases, the liver was the site of recurrence [11, 12, 14, 26]. Among them, only one patient was reported to have received chemotherapy for the treatment of recurrent liver tumors. The 49-year-old female patient had a recurrence of liver metastasis 12 months after surgery and received chemotherapy with gemcitabine for an additional 26 months. She subsequently underwent right liver lobectomy 39 months after the initial operation because of an increase in tumor size [26]. As the frequency of postoperative liver metastasis recurrence is high, patients must be closely followed up after pancreatectomy. However, few studies have reported the optimal chemotherapy regimen for patients with pure pancreatic HC. We identified two patients with metastatic pancreatic HC treated with sorafenib, an oral multikinase inhibitor approved for the treatment of advanced hepatocellular carcinoma [10, 27]. In these reports, sorafenib seemed to provide some short-term clinical benefits. Therefore, sorafenib may be a possible candidate for chemotherapy in patients with recurrent or unresectable pure pancreatic HC.

## Conclusions

We present a case of moderately differentiated pure pancreatic HC. Current clinical guidelines recommend biopsy as opposed to cytology or preoperative imaging studies for the preoperative diagnosis of pure pancreatic HC. While the natural history and prognosis of pure pancreatic HC may not be accurately predicted with these limited data, the prognosis of pure pancreatic HC could depend on the degree of differentiation.

## Data Availability

All data generated or analyzed during this study are included in this published article.

## Abbreviations

AFP: Alpha-fetoprotein
CEA: Carcinoembryonic antigen
CT: Computed tomography
EUS: Endoscopic ultrasound
FNA: Fine needle aspiration
HC: Hepatoid carcinoma
HepPar-1: Hepatocyte paraffin 1
MRI: Magnetic resonance imaging
PIVKA-II: Protein induced by vitamin K absence or antagonist-II
SPN: Solid pseudopapillary neoplasm

## References

[1] Ishikura H, Fukasawa Y, Ogasawara K, Natori T, Tsukada Y, Aizawa M (1985). An AFP-producing gastric carcinoma with features of hepatic differentiation. A case report. Cancer.

[2] Roberts CC, Colby TV, Batts KP (1997). Carcinoma of the stomach with hepatocyte differentiation (hepatoid adenocarcinoma). Mayo Clin Proc.

[3] Devi NR, Sathyalakshmi R, Devi J, Lilly SM (2015). Hepatoid adenocarcinoma of the gall bladder-a rare variant. J Clin Diagn Res.

[4] Søreide JA, Greve OJ, Gudlaugsson E, Størset S (2016). Hepatoid adenocarcinoma of the stomach- proper identification and treatment remain a challenge. Scand J Gastroenterol.

[5] Motooka Y, Yoshimoto K, Semba T, Ikeda K, Mori T, Honda Y (2016). Pulmonary hepatoid adenocarcinoma: report of a case. Surg Case Rep.

[6] Liu XL, Wang X, Zhu FF (2012). Hepatoid carcinoma of the ovary: a case report and review of the literature. Oncol Lett.

[7] Chen Y, Schaeffer DF, Yoshida EM (2014). Hepatoid adenocarcinoma of the colon in a patient with inflammatory bowel disease. World J Gastroenterol.

[8] Marchegiani G, Gareer H, Parisi A, Capelli P, Bassi C, Salvia R (2013). Pancreatic hepatoid carcinoma: a review of the literature. Dig Surg.

[9] Majumder S, Dasanu CA (2013). Hepatoid variant of pancreatic cancer: insights from a case and literature review. JOP.

[10] Petrelli F, Ghilardi M, Colombo S, Stringhi E, Barbara C, Cabiddu M (2012). A rare case of metastatic pancreatic hepatoid carcinoma treated with sorafenib. J Gastrointest Cancer.

[11] Kelly PJ, Spence R, Dasari BV, Burt AD, Taylor M, Loughrey MB (2012). Primary hepatocellular carcinoma of the pancreas: a case report and review of the heterogeneous group of pancreatic hepatoid carcinomas. Histopathology.

[12] Kai K, Nakamura J, Ide T, Masuda M, Kitahara K, Miyoshi A (2012). Hepatoid carcinoma of the pancreas penetrating into the gastric cavity: a case report and literature review. Pathol Int.

[13] Metzgeroth G, Ströbel P, Baumbusch T, Reiter A, Hastka J (2010). Hepatoid adenocarcinoma - review of the literature illustrated by a rare case originating in the peritoneal cavity. Onkologie.

[14] Yano T, Ishikura H, Wada T, Kishimoto T, Kondo S, Katoh H (1999). Hepatoid adenocarcinoma of the pancreas. Histopathology.

[15] Cardona D, Grobmyer S, Crawford JM, Liu C (2007). Hepatocellular carcinoma arising from ectopic liver tissue in the pancreas. Virchows Arch.

[16] Hughes K, Kelty S, Martin R (2004). Hepatoid carcinoma of the pancreas. Am Surg.

[17] Pellini Ferreira B, Vasquez J, Carilli A (2017). Metastatic hepatoid carcinoma of the pancreas: first description of treatment with capecitabine and temozolomide. Am J Med Sci.

[18] Hameed O, Xu H, Saddeghi S, Maluf H (2007). Hepatoid carcinoma of the pancreas: a case report and literature review of a heterogeneous group of tumors. Am J Surg Pathol.

[19] Paner GP, Thompson KS, Reyes CV (2000). Hepatoid carcinoma of the pancreas. Cancer.

[20] Seufferlein T, Bachet JB, Van Cutsem E, Rougier P (2012). ESMO Guidelines Working Group. Pancreatic adenocarcinoma: ESMO-ESDO Clinical Practice Guidelines for diagnosis, treatment and follow-up. Ann Oncol.

[21] Akimoto Y, Kato H, Matsumoto K, Harada R, Oda S, Fushimi S (2016). Pancreatic hepatoid carcinoma mimicking a solid pseudopapillary neoplasm: a challenging case on endoscopic ultrasound-guided fine-needle aspiration. Intern Med.

[22] Papavramidis T, Papavramidis S (2005). Solid pseudopapillary tumors of the pancreas: review of 718 patients reported in English literature. J Am Coll Surg.

[23] Kuo PC, Chen SC, Shyr YM, Kuo YJ, Lee RC, Wang SE (2015). Hepatoid carcinoma of the pancreas. World J Surg Oncol.

[24] Terracciano LM, Glatz K, Mhawech P, Vasei M, Lehmann FS, Vecchione R (2003). Hepatoid adenocarcinoma with liver metastasis mimicking hepatocellular carcinoma: an immunohistochemical and molecular study of eight cases. Am J Surg Pathol.

[25] Yang C, Sun L, Lai JZ, Zhou L, Liu Z, Xi Y (2019). Primary hepatoid carcinoma of the pancreas: a clinicopathological ctudy of 3 cases with review of additional 31 cases in the literature. Int J Surg Pathol.

[26] Matsueda K, Yamamoto H, Yoshida Y, Notohara K (2006). Hepatoid carcinoma of the pancreas producing protein induced by vitamin K absence or antagonist II (PIVKA-II) and alpha-fetoprotein (AFP). J Gastroenterol.

[27] Antonini F, Angelelli L, Rubini C, Macarri G (2015). Endoscopic ultrasound diagnosis of a primary hepatoid carcinoma of the pancreas. Endoscopy.

[28] Cuilliere P, Lazure T, Bui M, Fabre M, Buffet C, Gayral F (2002). Solid adenoma with exclusive hepatocellular differentiation: a new variant among pancreatic benign neoplasms?. Virchows Arch.

[29] Shih NN, Tsung JS, Yang AH, Tsou MH, Cheng TY (2006). A unique pancreatic tumor with exclusive hepatocytic differentiation. Ann Clin Lab Sci.

[30] Liu CZ, Hu SY, Wang L, Zhi XT, Jin B, Zhu M (2007). Hepatoid carcinoma of the pancreas: a case report. Chin Med J.

[31] Soofi Y, Kanehira K, Abbas A, Aranez J, Bain A, Ylagan L (2015). Pancreatic hepatoid carcinoma: a rare form of pancreatic neoplasm. Diagn Cytopathol.

[32] Steen S, Wolin E, Geller SA, Colquhoun S (2013). Primary hepatocellular carcinoma (“hepatoid” carcinoma) of the pancreas: a case report and review of the literature. Clin Case Rep.

[33] Vanoli A, Argenti F, Vinci A, La Rosa S, Viglio A, Riboni R (2015). Hepatoid carcinoma of the pancreas with lymphoid stroma: first description of the clinical, morphological, immunohistochemical, and molecular characteristics of an unusual pancreatic carcinoma. Virchows Arch.

[34] Veerankutty FH, Yeldho V, Tu SA, Venugopal B, Manoj KS, Vidhya C (2015). Hepatoid carcinoma of the pancreas combined with serous cystadenoma: a case report and review of the literature. Hepatobiliary Surg Nutr.

[35] Chang JM, Katariya NN, Lam-Himlin DM, Haakinson DJ, Ramanathan RK, Halfdanarson TR (2016). Hepatoid carcinoma of the pancreas: case report, next-generation tumor profiling, and literature review. Case Rep Gastroenterol.

[36] Kubota K, Kita J, Rokkaku K, Iwasaki Y, Sawada T, Imura J (2007). Ectopic hepatocellular carcinoma arising from pancreas: a case report and review of the literature. World J Gastroenterol.

[37] Stamatova D, Theilmann L, Spiegelberg C (2016). A hepatoid carcinoma of the pancreatic head. Surg Case Rep.

